# Injury rates in female and male military personnel: a systematic review and meta-analysis

**DOI:** 10.1186/s12905-022-01899-4

**Published:** 2022-07-25

**Authors:** Ben Schram, Elisa Canetti, Robin Orr, Rodney Pope

**Affiliations:** 1 Tactical Research Unit, Bond Institute of Health and Sport, Bond University, 2 Promethean Way, Robina, QLD 4229 Australia; 2 School of Community Health, Charles Sturt University, Albury, NSW Australia

**Keywords:** Women, Army, Defence, Tactical

## Abstract

**Background:**

An effective military force is required to be agile, capable, efficient, and potent. Injuries to military personnel interrupt active-duty service and can detract from overall capability. These injuries are associated with a high individual and organizational burden, with lost work time and financial costs—all problematic for the ongoing functioning of a military force. Injury control strategies have therefore been described as force multipliers. Female personnel form an integral part of any modern defence force, but little research has examined their specific experiences of injury, to inform targeted injury control efforts. The aim of this review was to identify and synthesise findings from studies of injury rates and patterns in female military personnel, comparing them to those of male personnel.

**Methods:**

A systematic search was conducted for studies which compared injury rates between the sexes at any stage of military service, from basic training through to deployment. Databases searched included PUBMED, CINAHL and Medline through OVID. Methodological quality of eligible articles was assessed using the Critical Appraisal Skills Program (CASP), and AXIS tools and data were extracted, synthesized, and, where possible, underwent meta-analysis.

**Results:**

Of 2287 identified studies, a total of 25 studies were eligible and included. Methodological quality ranged from 60% up to a perfect score of 100%, with an average of 82% across all studies. Relative risks for injuries (reported as RR [95%CI]) to females when compared to males were 2.10 [1.89–2.33] during basic training, 1.70 [1.33–2.17] during officer training, and 1.23 [1.05–1.43] post initial training. After adjustment for differences between the sexes in average fitness levels (2-mile run time), there was no longer a significant difference in injury rates (adjusted RR: 0.95 [0.86–1.05]). Female personnel tended to make bigger improvements in their fitness during basic training than males and tended to report their injuries more frequently and sooner than males.

**Conclusion:**

While this review found a higher rate of reported injuries in female military personnel when compared to male personnel, differences between the sexes in average fitness levels and injury reporting behaviours may largely explain this rate difference. The difference in rates of reported injuries was greatest during basic training, and reduced thereafter, possibly due in part to a reduced difference in fitness between the sexes or increased opportunity to self-determine workloads relative to fitness levels.

## Introduction

An effective military force is required to be agile, capable, efficient, and potent. Injuries to military personnel interrupt active duty service and detract from overall capability [1]. These injuries are associated with a high individual and organizational burden, with lost work time, financial costs and lost resources all problematic for the ongoing functioning of a military force. Injury minimization strategies have therefore been described as force multipliers [2], with basic training being a focus for many interventions, due to the higher reported rate of injury when compared to other times during military careers [3].

Female soldiers are an integral part of any modern defence force, with their contribution and involvement essential for mission success [4]. In recent times, combat related roles have become increasingly accessible for women, highlighting the importance of their role within military organisations [5]. There are several investigations which report that female military personnel have a propensity to be injured at a higher rate than male personnel both during training [6, 7] and on deployment [8, 9]. There are numerous biomechanical, anthropometric, anatomical, and physiological differences between men and women which may all contribute to differences in injury rates, body sites, and risk factors for injuries in military personnel. Some of the identified reasons for the disparity in injuries include biomechanical and anthropometric differences between the sexes. For example, female personnel on average have shorter leg lengths (anatomical structure), leading to over striding when marching in formation (biomechanical) when the pace is set by generally taller male personnel [10]. Other authors have postulated that anatomical differences such as bone geometry and mineralization of the tibia predispose female personnel to a higher incidence of injury, particular those classified as overuse injuries [11]. Differences in body site of injuries have also been observed, with the foot being injured more commonly during load carriage marches in female soldiers, as opposed to the ankle in male soldiers [12]. Likewise, integrated cohort training, where female and male personnel train together, has been highlighted as being a risk factor for injury in female personnel who show a higher cardiovascular strain during mixed training than male personnel [13].

Another reason for the potential disparity in injury rates between the sexes may be the way in which injury data are reported and collected. Data may vary substantially based on whether the injury reporting system involves self-reporting or adopts a point of care system, where details of injuries are captured during health care consultations [14]. Injury reporting systems which utilize a self-reporting method have been found to significantly under-estimate injury rates when compared to rates derived through point of care methods [14]. In addition, female personnel have been found to be more likely to report an injury and seek medical assistance than male personnel [15], with one study of US Marine Corps recruits in particular showing no difference in injury rates between sexes when both reported and non-reported injuries were pooled [16].

To date, there appears to be conflicting information on true injury rates, risk factors, and body sites of injury when female and male military personnel are compared. It is imperative that injury minimization strategies are informed by up-to-date, context specific, and evidence-based strategies which are relevant for, and potentially specific to, both sexes. Therefore, the aim of this review was to identify and synthesise findings from studies that have investigated compared injury rates in male and female military personnel throughout the military career span.

## Methods

A systematic review was conducted to address the research aim, guided by the Preferred Reporting Items for Systematic Reviews and Meta-Analysis (PRISMA) guidelines [17]. This review was registered with Prospero as part of a larger project (CRD42020170003). The tool ‘systematic review accelerator’ (sr-accelerator.com) was used when developing the final search strategy, to refine and optimise initial proposed search strategies and select databases. As most military personnel are male, and therefore most military research is focused on males, terms relating to female personnel were targeted to ensure that retrieved studies compared injury rates between the sexes. A search of three databases (PubMed, CINAHL and Medline through OVID) was conducted, using the search terms displayed in Table 1 in January 2020. Following removal of duplicates using Endnote software (Endnote X9, version X9.3.3, Clarivate Analytics, Philadelphia, United States), remaining articles were screened by title and abstract by two authors to remove articles which were clearly unrelated to the focus of this review and ineligible for inclusion. Any disagreements were settled with consultation with a third reviewer. Finally, remaining articles were scrutinised in full text to determine their eligibility based on the detailed criteria outlined below.

**Table 1:** Search terms used in each database

Database	Search terms
PUBMED	((((((female[Title/Abstract] OR women[Title/Abstract] OR woman[Title/Abstract])))) AND injur*[Title/Abstract])) AND ((defence[Title/Abstract] OR defense[Title/Abstract] OR military[Title/Abstract] OR army[Title/Abstract] OR "air force"[Title/Abstract] OR navy[Title/Abstract] OR marines[Title/Abstract] OR tactical[Title/Abstract] OR recruit[Title/Abstract] OR soldier[Title/Abstract] OR cadet[Title/Abstract] OR trainee[Title/Abstract]))
OVID MEDLINE	((((((female.ti,ab,kw. OR women.ti,ab,kw. OR woman.ti,ab,kw.)))) AND injur*.ti,ab,kw.)) AND ((defence.ti,ab,kw. OR defense.ti,ab,kw. OR military.ti,ab,kw. OR army.ti,ab,kw. OR air force.ti,ab,kw. OR navy.ti,ab,kw. OR marines.ti,ab,kw. OR tactical.ti,ab,kw. OR recruit.ti,ab,kw. OR soldier.ti,ab,kw. OR cadet.ti,ab,kw. OR trainee.ti,ab,kw.))
CINAHL	((((((TI female OR AB female OR SU female OR TI women OR AB women OR SU women OR TI woman OR AB woman OR SU woman)))) AND TI injur* OR AB injur* OR SU injur*)) AND ((TI defence OR AB defence OR SU defence OR TI defense OR AB defense OR SU defense OR TI military OR AB military OR SU military OR TI army OR AB army OR SU army OR TI "air force" OR AB "air force" OR SU “air force” OR TI navy OR AB navy OR SU navy OR TI marines OR AB marines OR SU marines OR TI tactical OR AB tactical OR SU tactical OR TI recruit OR AB recruit OR SU recruit OR TI soldier OR AB soldier OR SU soldier OR TI cadet OR AB cadet OR SU cadet OR TI trainee OR AB trainee OR SU cadet))

Studies were included if they: (a) reported on injury rates, with comparisons between male and female personnel; (b) were conducted in the context of military training, service, or deployment; and (c) investigated injuries in general, rather than only specific types of injuries. Intervention studies were only included if they reported injury rates separately for men and women in their control group—rates from personnel in experimental or intervention groups were not included.

Studies were excluded if: (a) they did not report injury rates for personnel of both sexes; or (b) they reported only on a specific injury type (e.g., stress fracture of the tibia), or body site (e.g., lower limb injuries), or level of severity of injury (e.g., injuries which resulted in hospitalisation, fatalities or time loss), or (c) solely focused on combat injuries. Also excluded were articles which reported injury rates without reporting underlying cohort size data from which these were calculated or reported injury data that were not separable from other medical conditions or occupational performance outcomes. Studies of specialist training or procedures (e.g., parachuting or military police training) were also not included as they were deemed to not be representative of training that most military personnel would typically undertake. Finally, articles which did not report primary research, abstracts, articles for which full text could not be obtained, study protocols, and articles published in languages other than English and several other languages the research team could translate were also excluded.

The eligibility criteria were purposely broad, as studies which did not have a primary aim of comparing injury rates between sexes may still have reported injury rates as additional findings.

The methodological quality of each included study was appraised using the Critical Appraisal Skills Program (CASP) [18] tool for cohort studies and the AXIS tool for cross sectional studies [19] by two reviewers independently. The CASP tool has 12 questions and a maximum possible score of 12, with both questions 5 and 6 containing two sections, but questions 7 and 8 not being scored, due to their subjectivity. The AXIS has 20 questions and a total possible score of 20. The raw scores from each tool were converted to percentage scores, whereby the quality rating assigned to scores < 45.4% was ‘poor’, 45.4–61.0% ‘fair’, and > 61.0% ‘good’ [14]. The methodological quality score for each study was included in the key data table, to allow for the data extracted from each study to be considered in context of the methodological quality of the study.

Key data from eligible studies were then extracted and tabulated by two independent reviewers. The key data included: author(s) and year of publication; the military cohort studied and their country; the type of training and duration of data capture; the aim of the study; the injury identification method; and whether any adjustments were made in data analyses, for fitness levels of participants.

### Statistical analyses

Where the form of data allowed, available data were entered into Review Manager (RevMan, version 5.4) to calculate comparative statistics such as odds ratios, relative risks and 95% confidence intervals. Injury incidence rates per 1000 soldier-years of military service were also calculated where data allowed, whereby the number of injuries reported was divided by the cohort size before the resulting figure was then divided by the number of full-time equivalent years (i.e., cumulative periods of 365 days) each participant served and was followed during the study period and then multiplied by 1000. To enable valid comparisons between studies, for those studies which reported incidence rates per person-year of exposure, incidence rates were recalculated from the cohort size and number of injured personnel, as some studies used 365 days for an annual exposure, some 232 days (estimated working days in a year), and some a number of days that was not defined or stated, when calculating incidence rates. For studies which reported incidence rates but did not state the raw number of injuries, the same formula was used, first solving for the number of injuries based on stated incidence rate, period of follow-up and cohort size.

Data from included studies were further analysed through meta-analyses where possible using the Cochrane Collaboration’s Review Manager (RevMan, Version 5.3) software package. Given the differences in intensity, hours of exposure, and duration of training or deployments, studies were analysed by setting type, being basic training, advanced individual training, and enlisted, active duty or deployed personnel.

## Results

**Fig. 1:**
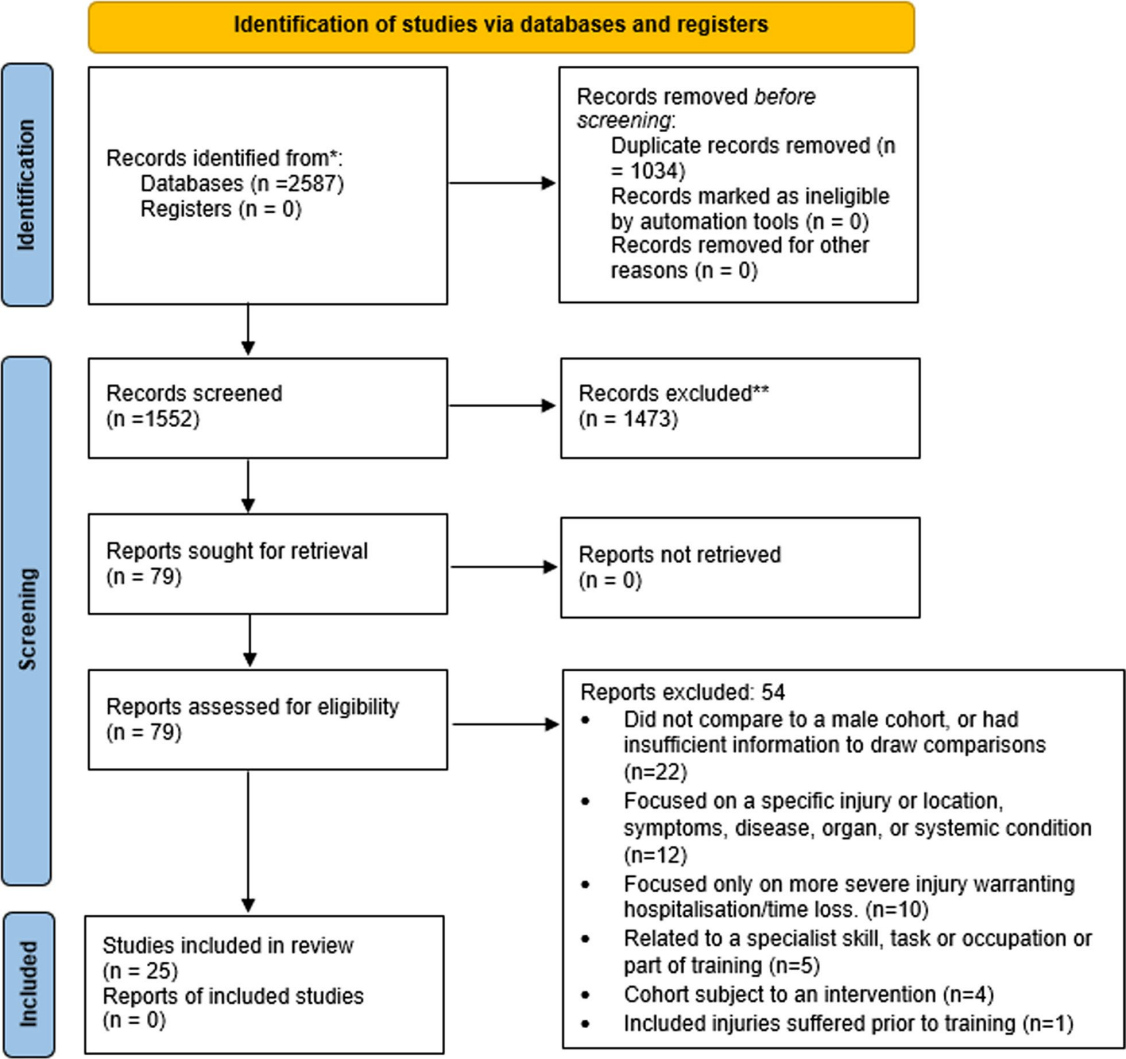
Prisma diagram depicting results of the search, screening and selection processes

From the initial search, a total of 2587 articles were identified, from which 1035 duplicates were removed, leaving 1552 articles for further screening (Fig. 1). After screening by titles and abstracts, 79 articles remained and were examined in full text. Finally, 25 studies were deemed eligible for inclusion in the review. Reasons for exclusion of articles examined in full text can be found in Fig. 1.

Included studies were mainly cohort studies (n = 19) [6, 20–37]. The remaining studies included five cross sectional studies [4, 38–41], and one case control study [42]. Methodological quality ranged from 60% [4] to a perfect score of 100% [22], with an average of 82% across all study types. Key data from each included study are presented in Table 2.

**Table 2:** Key data from included studies

References	Population	Reporting method	Factors for which findings were adjusted	Key findings	Injury incidence rate/1000/year*	RR (♀:♂)	Critical appraisal score
Anderson [4]	Active-Duty US Army (363 females, 4384 males) over 12 months	Self-reported survey	Gender, age, BF, smoking, fitness physical demand	Injury incidence over 12 months			
				♀ = 53% ♂ = 42%	♀ = 530/1000/year ♂ = 420/1000/year	1.26 [1.14–1.40] *p* < 0.01) aRR = 0.95 [0.86–1.05]	60%
Anderson [20]	Active-Duty US Army (43 women, 727 men) over 12 months	Self-reported survey	None	Injury rate Pre-deployment			
				♀ 42.6/1000/month ♂ 36.2/1000/month	♀ = 511.2/1000/year ♂ = 434.5/1000/year	1.18 [0.87–1.59] *p* = 0.33	83%
				On-deployment			
				♀ 14.0/1000/month ♂ 19.0/1000/month	♀ = 168.0/1000/year ♂ = 228.0/1000/year	0.71 [0.36–1.42] *p* = 0.33	
Bell [21]	Trainees undergoing US Army Basic Training over 8 weeks (352 women, 509 men)	Medical records	Fitness, age and race	Injury rate over 8 weeks of basic training			
				♀ = 57% ♂ = 27%	♀ = 3721.8/1000/year ♂ = 1767/1000/year	2.1 [1.78–2.47] aRR = 1.14 [0.48–2.72]	92%
Bijur [6]	558 West Point Officer Cadets in 6 weeks of basic training and initial 6 months of all training (85 women, 473 men)	Medical records	Fitness	Injury rate in 6 weeks of basic training			
				♀ = 67/85 cadets 78.8/100 cadets ♂ = 152/473 cadets 32.1/100 cadets After adjusting for fitness, difference between males and females was only 20/100 cadets, as opposed to 46.7/100 previously (including multiple injuries)	♀ = 6829.3/1000/year ♂ = 2782.0/1000/year	2.45 [2.06–2.91] *p* < 0.001	92%
				By end of 1st semester (subsequent 20 weeks of training)			
				♀ = 47.5/100 cadets 4 ♂ = 40.5/100 cadets (*p* = 0.42)	♀ = 1235/1000/year ♂ = 1053/1000/year	1.16 [0.90–1.49] *p* = 0.25	
				End of 2nd semester (2nd 6-month period of training)			
				♀ = 32.3/100 cadets ♂ = 38.8/100 cadets (*p* = 0.42)	♀ = 646/1000/year ♂ = 776/1000/year	0.82 [0.59–1.14] *p* = 0.23	
Billings [23]	Recruits in US Air Force Academy Basic Training over 12 months (224 women, 986 men)	Combination of 3 systems	None	Injury incidence per annum			
				♀ = 1067 per 1000 cadets ♂ = 615.6/1000 cadets No difference in number of restricted duty days	♀ = 1067/1000/year ♂ = 615.6/1000/year	1.73 [1.67–1.80]	75%
Blacker [22]	Recruits in British Army Basic Training over 12 weeks (1480 females, 11,937 males)	Medical records	Fitness	Injury incidence over 12 weeks basic training			
				♀ = 0.173/100 person days (202/1480) ♂ = 0.061/100 person days (550/11,937) HR 2.91 [2.48–3.43] *p* < 0.001 Sex did not feature in the multivariate model predicting injury risk. 2.4 km run time was a significant risk factor for injury (*p* < 0.001)	♀ = 631.5/1000/year ♂ = 222.7/1000/year	2.96 [2.55–3.44] *p* < 0.001	100%
Cosio-Lima [24]	Active-duty US Army soldiers over 9.5 months (6 females, 143 males)	Medical records	None	Injury incidence in 9.5 months			
				♀ = 33% ♂ = 50.3%	♀ = 422.6/1000/year ♂ = 644.8/1000/year	0.66 [0.21–2.08] *p* = 0.48	75%
Darakjy [25]	Active-Duty US Armor Division soldiers during 37 days of continuous training (413 females, 4101 males)	Medical records	None	Injury rate in 37 days			
				♀ = 22.9/1000 soldiers/week ♂ = 11.8/1000 soldiers/week	♀ = 1190.8/1000/year ♂ = 613.6/1000/year	1.93 [1.54–2.32] *p* < 0.001	83%
Fadum [38]	Active-Duty Norwegian Military Women over 12 months (1068 females, 8100 males)	Self-reported survey	None	Injury rates over 12 months			
				♀ = 284/1068 (26.6%) ♂ = 2034/8100 (25.1%)	♀ = 265.9/1000/year ♂ = 251.1/1000/year	1.06 [0.95–1.18] *p* = 0.29	85%
Grier [26]	Trainees at US Army Ordinance school with courses ranging from 9–16 weeks (498 females, 3757 males)	Self-reported survey and medical records (prospective and retrospective)	* Stratified	Injury rate for 9–16 weeks training			
				♀ = 61% ♂ = 36%		1.70 [1.56–1.84] *p* < 0.001	83%
Havenetidis [27]	Greek Army Officer Cadets in training for 7 weeks (20 females, 233 males)	Physician recorded	None	Injury rate during 7 weeks of training			
				♀ = 35% ♂ = 31.7%	♀ = 2607.1/1000/year ♂ = 2365.8/1000/year	1.10 [0.59–2.06] *p* = 0.76	67%
Henderson [39]	US Army Recruits undergoing Basic Training for 8 weeks (237 females, 371 males) and trainees in US Combat Medics AIT (10 week) (287 females, 439 males)	Medical records	None	BCT cumulative injury incidence	BCT		
				♀ = 51.5% (122/237) ♂ = 26.1% (97/371)	♀ = 3355.2/1000/year ♂ = 1704.1/1000/year	1.97 [1.59–2.43] *p* < 0.001	75%
				AIT cumulative injury incidence	AIT		
				♀ = 29.6% (85/287) ♂ = 23.7% (104/439)	♀ = 1544.3/1000/year ♂ = 1235.3/1000/year	1.25 [0.98–1.60] *p* = 0.078	
Jones [28]	Recruits in US Army Basic Training for 8 weeks (186 females, 124 males)	Medical records	None	Injury rates			
				♀ = 50.5% ♂ = 27.4%	♀ = 3294.0/1000/year ♂ = 1787.2/1000/year	1.84 [1.34–2.54] *p* < 0.001	83%
Jones [29]	Recruits in US Army Basic Training for 8 weeks (41,727 females, 143,398 male)	Medical records	None	Injury rates			
				♀ = 40.3% ♂ = 15.7%	♀ = 2626.7/1000/year ♂ = 1023.3/1000/year	2.57 [2.52–2.61] *p* < 0.001	83%
Kerr [30]	Recruits in Irish Army Basic Training for 16 weeks (40 females, 354 males)	Medical records	None	Injury incidence			
				♀ = 99.26 injuries/1000-man weeks ♂ = 56.96 injuries/1000-man weeks	♀ = 5161.5/1000/year ♂ = 2961.9.1/1000/year	1.76 [1.06–2.94]	75%
				Injury rates			
				♀ = 54/40 ♂ = 271/354			
Knapik [33]	Recruits in US Army basic training for 8 weeks (Summer: 434 females, 733 males) (Fall: 591 females, 810 males)	Medical records	None	Summer injury rates	Summer		
				♀ = Summer 63.1% ♂ = Summer 37.0%	♀ = 4115.0/1000/year ♂ = 2409.7/1000/year	1.71 [1.52–1.92] *p* < 0.001	92%
				Fall injury rates	Fall		
				♀ = 44.8% ♂ = 18.9%	♀ = 2922.6/1000/year ♂ = 1231.2/1000/year	2.37 [2.00–2.81] *p* < 0.001	
				Total injury rates			
				♀ = 539/1025 ♂ = 424/1543	♀ = 3427.4/1000/year ♂ = 1791.0/1000/year	1.91 [1.73–2.11] *p* < 0.001	
Knapik [31]	Recruits in US Army basic training for 8 weeks (247 females, 567 males)	Medical records	None	Injury rates for ‘control group’			
				♀ = 1.07/100 person days (148/247) ♂ = 0.56/100 person days (178/567)	♀ = 3905.4/1000/year ♂ = 2046.2/1000/year	1.91 [1.63–2.24] *p* < 0.001	83%
Knapik [32]	Recruits in US Army Basic Training for 8 weeks) (452 females, 733 males)	Medical records	None	Injury incidence rates			
				♀ 1.16/100 person days ♂ 0.56/100 person days	♀ = 4234.0/1000/year ♂ = 2044.0/1000/year	2.07 [1.83–2.35] *p* < 0.001	92%
Kovcan [40]	Active-Duty Slovenian Armed Forces over one year. (11 females, 118 males)	Injury report form	None	Prevalence			
				♀ = 27.7% ♂ = 50.8%	♀ = 272.7/1000/year ♂ = 508.5/1000/year	0.54 [0.20–1.43] *p* = 0.21	75%
Nye [34]	Active-Duty USAF monitored over 7 years. (6398 females, 61,506 males)	Database	None	Injury rates over 7 years			
				♀ = 71.8% (4597/6398) ♂ = 67.1% (41,278/61,506)	♀ = 102.6/1000/year ♂ = 95.9/1000/year	1.07 [1.05–1.09] *p* < 0.001	92%
Nye [41]	Recruits in USAF Basic Training (8.5 week) 14,550 females, 52,975 males)	Database	None	Overall incidence rate in 8.5 weeks training			
				♀ = 29.4 [28.6–30.3] injuries /1000 person weeks (2862/14550) ♂ = 15.1 [14.7–15.4] injuries/1000 person weeks (5586/52975)	♀ = 1196.6/1000/year ♂ = 641.5/1000/year	1.87 [1.79–1.94] *p* < 0.001	80%
Piantanida [35]	Cadets in US Marines officer Basic training for 6 weeks. (30 females, 450 males)	Medical records	None	Cumulative injury incidence in 6 weeks			
				♀ = 80% ♂ = 59.5%	♀ = 6952.4/1000/year ♂ = 5175.7/1000/year	1.34 [1.11–1.63] *p* < 0.003	75%
Snedcor [36]	Recruits in US Airforce Basic Training for 30 training days. (5250 females, 8660 males)	Outpatient visits	None	Injury rate over basic training			
				♀ = 63.0/1000 person weeks [60.6–65.5] (1743/5250) ♂ = 27.8/1000 person weeks [26.4–29.2] 1329/8660)	♀ = 3276.0/1000/year ♂ = 1445.6/1000/year	2.16 [2.03–2.30] aRR = 2.22 [2.09–2.37]	83%
Strowbridge [37]	Active-Duty British Army Personnel over 1 year (178 females, 3377 males)	Medical records	None	Injury rate over 1 year			
				♀ = 44.5/1000/month ♂ = 26.6/1000/month	♀ = 534.0/1000/year ♂ = 319.2/1000/year	1.67 [1.45–1.93] *p* < 0.001	67%
Sulsky [42]	Recruits in US Army Basic Training for 8 weeks. (21,651 females, 139,020 males)	Medical records	None	At least one injury in basic training			
				♀ = 61% (16,833/21,651) ♂ = 39% (54,784/139,020)	♀ = 5067.4/1000/year ♂ = 2568.5/1000/year	1.97 [1.95–1.99] *p* < 0.001	91%

RR, relative risk; aRR, adjusted relative risk; BF, body fat; N/S, not significant

*Calculated injury incidence rate per 1000 personnel per year

The studies were from a range of countries, with 19 from the United States [4, 6, 20, 21, 24–26, 28, 29, 31–36, 39, 41, 42], two from both the United Kingdom [22, 37] and one each from Norway [38], Greece [27], Ireland [30], and Slovenia [40]. Army was the most represented service, accounting for 18 studies [4, 6, 20–22, 24–33, 37, 39, 42], followed by Air Force, with four studies [23, 34, 36, 41]. Two studies were of the Armed Services more broadly [38, 40] and the remaining study involved Marines [35]. Investigations were most often conducted in basic training contexts, accounting for 14 of the studies [21–23, 28–33, 36, 41, 43–45], followed by active duty personnel for eight studies [4, 20, 24, 25, 34, 37, 38, 40], officer training for three studies [6, 27, 35], and Advanced Individual Training (AIT) or Initial Employment Training (IET) for two studies [26, 39]. All of the training performed in contexts outside the United States occurred in mixed sex training programs [27, 30, 37, 38, 43].

The most common method of obtaining injury data in the included studies was by reviewing medical records (n = 19), while some studies used self-report surveys [4, 20, 38], or a mixture of methods [23, 26, 40]. Only four studies accounted for fitness levels when comparing injury rates between the sexes [4, 6, 21, 22].

### Injury rates

A total of 20 of the included studies reported that female personnel were injured at a higher rate than male personnel. One study reported that being deployed on operations was associated with lower injury rates for both sexes than pre-deployment training, however a significantly greater injury rate was found amongst female personnel when compared to male personnel, pre-deployment, while rates were similar during deployment [20]. Four studies reported that there were no differences in injury rates between male and female personnel [24, 30, 38, 40]. Two studies [6, 39] followed personnel through to later in their initial training, observing injury rates of officer cadets either at the end of their first semester and then second semester [6], or recruits after initial training and during Advanced Individual Training [39], and found there were no significant differences in injury rates between female and male personnel during those later training stages, despite initial higher rates in female personnel.

**Fig. 2:**
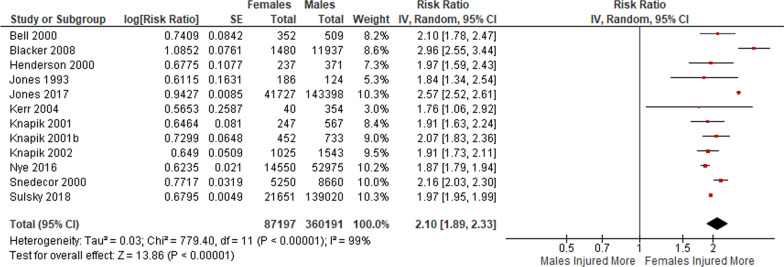
Meta-analysis of injury rates in basic training

A total of 12 studies reported on injury rates during basic training. A meta-analysis of key findings from these studies (Fig. 2) yielded a higher incidence rate of injury during basic training in female personnel than in males (RR = 2.10 [95% CI 1.89–2.33]), however with a high level of heterogeneity across the 12 studies (I^2^ = 99%).

**Fig. 3:**
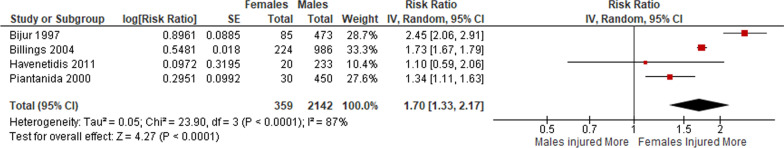
Meta-analysis of injury rates in officer training

Four studies reported on injury rates during officer training, including at the US Military Academy at West Point [6], the US Air Force Academy [23], the Greek Hellenic Army Academy [27], and Marine Corps Officer training [35]. Cadets were monitored for a duration ranging from six weeks [6, 35] to one year [23]. Meta-analysis of the four studies (Fig. 3) found a higher incidence rate of injury amongst female personnel, with a RR of 1.70 [95% CI 1.33–2.17].

**Fig. 4:**
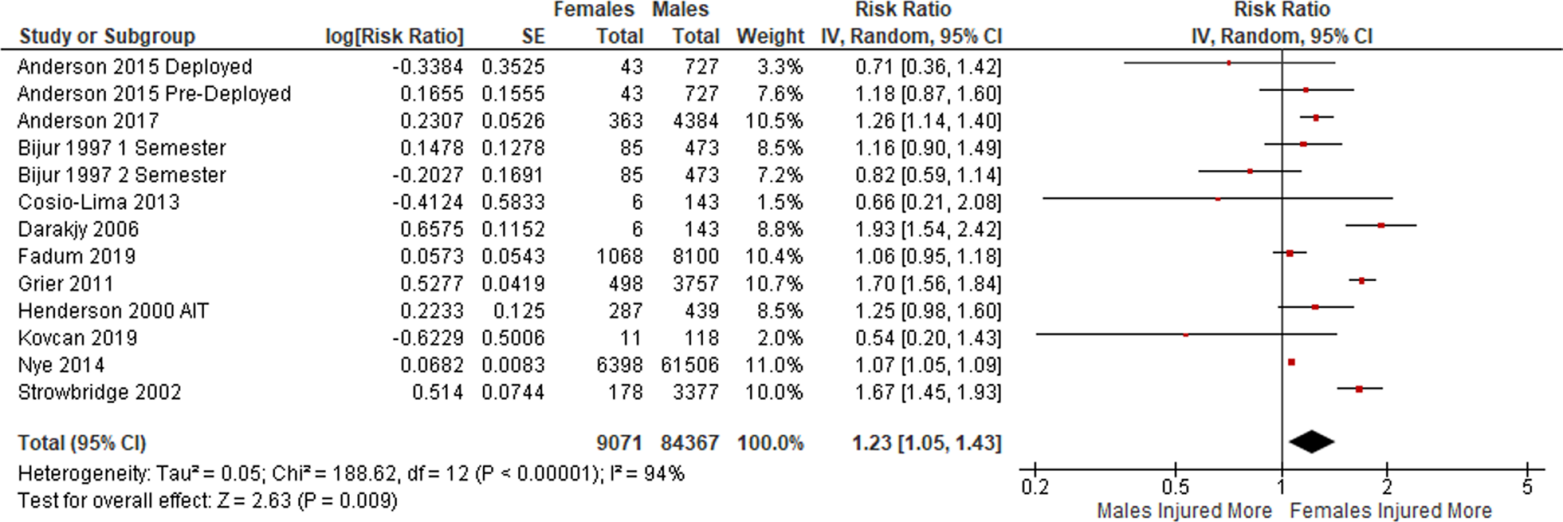
Meta-analysis of injury incidence rates post basic training

Eleven articles reported on injury rates after the completion of basic training, including during AIT [26, 39], at the latter stages of training at the US Military Academy [6], during a Sergeant Majors’ course [24], in active duty United States military personnel [4] and British Army personnel [37], in a US armour division [25], in active duty personne in the US Air Force l [34] and Norwegian [38] and Slovenian Armed Forces [40], and both pre and during deployment in the study by Anderson et al. [20]. Figure 4 shows a meta-analysis of these studies, which found the overall injury incidence rate was higher in female personnel than male personnel (RR = 1.23 [95% CI 1.05–1.43]).

**Fig. 5:**
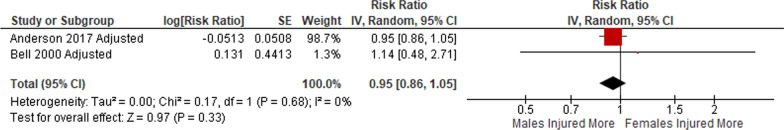
Meta-analysis of injury incidence rates from studies which adjusted for fitness

Of the articles which took fitness into account, three of the four [4, 21, 22] found that differences between the sexes in injury incidence rates were no longer significant once adjusted for fitness levels. However, the fourth study, despite finding the difference in injury rates between sexes decreased substantially, still showed a significantly greater injury incidence rate among female cadets [6]. Both the study by Anderson et al. [4] and that by Bell et al. [21] provided risk estimates which were combined in a meta-analysis (Fig. 5), which in turn found no difference in injury rates between female and male personnel (RR = 0.95 [95% CI 0.86–1.05]). Despite not providing adjusted risk estimates for each sex, the study by Blacker et al. [22] found that gender did not feature in a multivariate regression model which explored risk factors for injury, while fitness level did.

## Discussion

The aim of this review was to identify and synthesise findings from studies that have investigated and compared injury rates in male and female military personnel throughout the military career span. The volume of evidence from the included studies suggests that female military personnel are injured at a higher rate than male personnel in military populations. However, there are confounding variables which were not adjusted for consistently, including fitness, mixed training approaches, and reporting methods. The point of time in a military career at which comparisons were made also appears to be an important factor, with a large difference in injury rates between the sexes found during basic training, which appears to decrease substantially during subsequent employment training and later in careers.

Despite the evidence of an elevated injury rate among female military personnel when compared to male personnel, studies which have performed multivariate analysis have found differing results, suggesting that any observed differences between the sexes in injury rates may not be due to biological sex, per se, but to other factors which on average differ between the sexes. For example, adjustment of injury incidence rates for level of physical fitness [4, 21, 22] resulted in no difference in injury rates being found between sexes and it is well known that average fitness levels in female new recruits are lower than average levels in male new recruits [21]. Despite most of the investigations involving United States military personnel, military forces from other countries have reported differing results, with British [46] and Slovenian [40] armed forces displaying a higher injury rate among male personnel, and Norwegian military personnel observed to have similar musculoskeletal injury rates between the sexes [38]. Likewise, evidence to date indicates male military personnel may be at a higher risk of more serious injury in some contexts [47], especially while on deployment [48, 49] and with regard to specific injuries, such as spinal cord injury or scaphoid fracture [50, 51].

Unadjusted injury rates appear to be higher for female personnel during basic training when compared to their male counterparts. This difference is not as large between the sexes during officer training and is smaller again post initial training (during advanced individual training) and at subsequent stages of military careers. The lower injury rate difference between the sexes in officer training may be explained by the typically shorter duration of some officer basic training courses (6–7 weeks officer training vs 8–12 weeks basic training) or by the fact there are fewer studies which have focused on officer training. Entry as an officer is often governed by both academic and fitness standards [23], which may also play a role in the observed differences in injury rates.

The decrease in injury rate differences at latter stages of military training may be due to increases in fitness of female personnel due to military training itself, or through ‘survivor’ bias, where those who are at higher injury risk and thus injured may have been discharged and therefore no longer remain within the training population. For example, having completed an entire semester of physical training similar to that undertaken by male soldiers, female soldiers no longer had a significantly different injury rate [6]. The reason proposed for this lack of difference was the physical fitness between the sexes being more similar, subsequent to initial training completion [6]. This levelling of injury risk was also seen at latter stages of training, in the study by Henderson [39], where despite a significantly higher injury risk for women during basic training, injury risks in the subsequent Advanced Individual Training were similar between the sexes.

Despite the decrease in injury risk for female personnel relative to male personnel following military training, there is some debate as to whether female personnel training with male personnel is a risk factor for injury in itself [52, 53]. Studies on mixed platoons in training have shown that the intensity of training measured by heart rate, ratings of perceived exertion, or energy expenditure is typically much greater for female personnel than their male cohort counterparts [13, 54], most likely due to the lower average fitness levels of female personnel. This increased intensity has been thought to place female personnel at a greater risk of injury due to the increase in physiological strain [13]. However a recent investigation found that, despite female trainees having a higher internal training load as measured by time in heart rate zones and training impulse, the overall injury rate was not significantly different between the sexes in a mixed platoon, albeit within a small sample size of a single platoon (n =  ~ 30) [46].

Additionally, the benefit to fitness levels for females training with males needs to be considered. A study by Bell [21] found that, despite male trainees still being able to perform more push-ups and complete runs faster, on average, than female trainees at the end of basic training, female trainees achieved greater improvements in sit-ups, push-ups and run times than male trainees. This greater improvement in performance is suggested to be due to females, in general, entering military training less physically fit relative to their training potential when compared to males [21]. Finding the balance between an intensity which is adequate for improvement in fitness for both sexes, without a concurrent increase in injury risk, is paramount, but a difficult task.

Most studies considered in this review concluded that female personnel are injured at a higher rate than male personnel. This finding should be interpreted with caution, however, as adjusting for fitness—a modifiable risk factor—appears to substantially decrease this difference. Four studies adjusted injury rates for fitness levels, and three found no differences in injury risk between the sexes [4, 21, 22]. The fourth found a 50% reduction in the difference in injury rates between women and men, however the difference still remained significant [6].

Likewise, there is some evidence to suggest the method of injury reporting and differences between the sexes in reporting behaviours may explain some of the sex-related differences in injury rates [15]. For example, female personnel are purported to be more likely to seek medical care for injuries than their male counterparts [6] and so may be perceived to have higher injury rates simply because they report more of the injuries they suffer. Consequently, if differences in injury rates can be largely explained by average differences between the sexes in fitness levels, the additional effect of reporting behaviours may mean that male personnel are actually injured at a higher rate than female personnel. This supposition may be supported by the findings in British [46] and Slovenian armed forces [40], which show an elevated injury rate among male personnel. It is therefore advised that future studies which examine injury rates between the sexes adjust for both fitness and reporting methods and behaviours, to enable accurate attributions for any observed differences in rates of injury between the sexes.

Limitations to this review include the various methods of reporting of comparative levels of risk in the included studies, such as with odds ratios, risk ratios, or proportions of individuals who were injured. This led to many risk ratios being calculated or converted from reported odds ratios and sex-specific proportions, which may lead to rounding errors. Sample sizes for female personnel were typically small, highlighting how underrepresented female personnel are within the research literature. Additionally, there are few large studies conducted on military personnel outside of the United States, and so it is difficult to gauge the extent to which the findings from this review are generalisable to military forces of other nations. Additionally, variations in what was considered to constitute an injury existed across studies, with some focusing more on time loss injuries, some more on injuries which led to discharge, and some capturing all injuries. It was the intent of this review to capture all injuries, not just more severe injuries which led to lost time from military service or admission to hospital, and the inclusion of studies which used these sorts of limited injury definitions may have affected the findings of the review.

## Conclusion

Females in military service appear to be injured at higher rates than their male counterparts, particularly during basic training. The difference in injury rates decreases after basic training, culminating in minimal differences at latter stages of military careers. However, some and perhaps the majority of these differences may be due to average differences between the sexes in fitness levels, particularly early in a military career. Additionally, some reported differences may not represent true differences in injury rates but rather a propensity for female personnel to more frequently report injuries they have suffered than their male counterparts. These confounding factors mean that observed sex differences in injury rates are unlikely to be due to biological sex, per se, but rather to average sex-based differences in fitness levels, reporting behaviours and possibly other factors. Taking this further, the influence of these other factors may mean that, when fitness levels, as a modifiable risk factor, are equalised, in some contexts male military personnel may actually experience higher rates of injury than female personnel. Strategies to increase the fitness of female military personnel prior to them commencing basic training need to be developed.

## Data Availability

All data generated or analysed during this study are included in this published article or from the articles included in this review.

## Abbreviations

AIT: Advanced individual training
aRR: Adjusted relative risk
BCT: Basic combat training
BF: Body fat
CASP: Critical Appraisal Skills Program
CI: Confidence interval
HR: Hazard ratio
IET: Initial employment training
IR: Incident rate
IRR: Incident rate ratio
OR: Odds ratio
RR: Relative risk
US: United States
USAF: United State Air Force
